# The mitogenome of the scale insect *Didesmococcus koreanus* Borchsenius, 1955 (Coccoidea: Coccidae)

**DOI:** 10.1080/23802359.2021.1906175

**Published:** 2021-04-04

**Authors:** Han Xu, Xiaochen Liu, Hu Li, Sanan Wu

**Affiliations:** a The Key Laboratory for Silviculture and Conservation of Ministry of Education, Beijing Forestry University, Beijing, PR China; b Department of Entomology, College of Plant Protection, MOA Key Lab of Pest Monitoring and Green Management, China Agricultural University, Beijing, PR China

**Keywords:** Coccidae Mitochondrial genome gene rearrangement

## Abstract

The complete mitochondrial genome of *Didesmococcus koreanus* is described in this study. The complete mitogenome is a 15,162 bp circular DNA molecule with a high A + T content of 82.5%, containing the typical 37 genes. A novel gene rearrangement is detected in the mitogenome. The phylogenetic result supports the monophyly of Coccoidea and the closer relationship between the Ceroplastinae and Coccinae.

The Coccidae, as the third largest family in the superfamily Coccoidea, contains more than 1 200 species belonging to the 174 genera, widely distributed around the world. And many coccid species are economically significant insects in agriculture, horticulture, and forestry ( Miller and Davidson 1990; Miller et al. 2005). But understanding of the phylogenetic relationships within this family remains limited because the taxonomy on the Coccidae is based largely on the morphological characters from the female adult (Gullan and Cook 2007; Choi and Lee 2020). In this study, the complete mitochondrial genome of *Didesmococcus koreanus* Borchsenius, 1955 was reported and the phylogenetic relationship was reconstructed combined with other two coccid species and other 17 hemipteran species (Deng et al. 2019; Lu et al. 2020).

The 11 specimens were collected in Qinghua East Road No. 17, Haidian District in Beijing, China (the east campus of China Agricultural University) (116.35832°E, 40.00433°N). The specimens were deposited in the Insect Collection at the Department of Forestry Protection, Beijing Forestry University, Beijing, China (BFUC) (please contact Dr. Han Xu, email: xuhan@bjfu.edu.cn) under the voucher number cdk2016060601. The whole bodies of six specimens were used for the genomic DNA extraction with the TIANamp Genomic DNA Kit under the manufacturer’s instructions. The mitogenome was sequenced by Illumina Hiseq 2500 with 250 bp paired-end reads at Berry Genomics (Beijing, China). The reads were assembled with IDBA-UD (Peng et al. 2012) and then assembled mitogenome was annotated with the MITOs WebServer (Bernt et al. 2013) and manual method with reference to other hemipteran species. And the sequence was deposited in GenBank under the accession number MW302211.

The complete mitogenome is a circular DNA molecule with 15,143 bp in size and contains entire sets of 37 genes, including 13 protein-coding genes (PCGs), 22 tRNA genes, 2 rRNA genes, and one control region. The gene arrangement differs from that of the putative ancestor of insects but similar to species *Saissetia coffeae* (Walker 1852) except for the translocation between the *trnC* and *trnA* (Boore 1999; Lu et al. 2020). The nucleotide composition of the whole mitogenome is significantly biased toward A + T (82.5%). All PCGs initiate with ATN as the start codon (three with ATA, two with ATG, and eight with ATT). The typical stop codon TAA was adopted by eight PCGs and TAG occurs in four PCGs, e.g. *cytb*, *nad1*, *nad4L*, and *nad6*, except that only *nad4* terminates with a single T residue.

All 22 tRNA genes were detected, only ten of which exhibited the classic cloverleaf secondary structure. The other 12 tRNA genes were truncated. The *trnE*, *trnG*, *trnH*, and *trnR* lacked the TΨC arm. The *trnL1*, *trnQ*, *trnS1*, and *trnY* lost the DHU arm. And the *trnA*, *trnS2*, *trnT*, and *trnW* lacked both DHU and TΨC arms. *rrnL* and *rrnS* both showed the high AT bias with A + T content of 86.7% and 85.3%, respectively. The control region is 1350 bp with a significant AT-bias with 77.7%.

The phylogenetic relationship was reconstructed based on the 13 protein-coding genes and two rRNA genes from 20 species using the Phylobayes MPI on XSEDE with the Bayesian method under GTR + CAT model (Figure 1) (Li et al. 2017; Liu et al. 2018). The result showed that the monophyly of Coccoidea was highly supported and *Ceroplastes japonicus* Green, 1921 was the sister group of *S. coffeae*. This may imply the Ceroplastinae and Coccinae have a closer relationship related to the Eulecaniinae represented by *D. koreanus*, consistent with the current result based on multiple molecular markers (Choi and Lee 2020).

**Figure 1.**
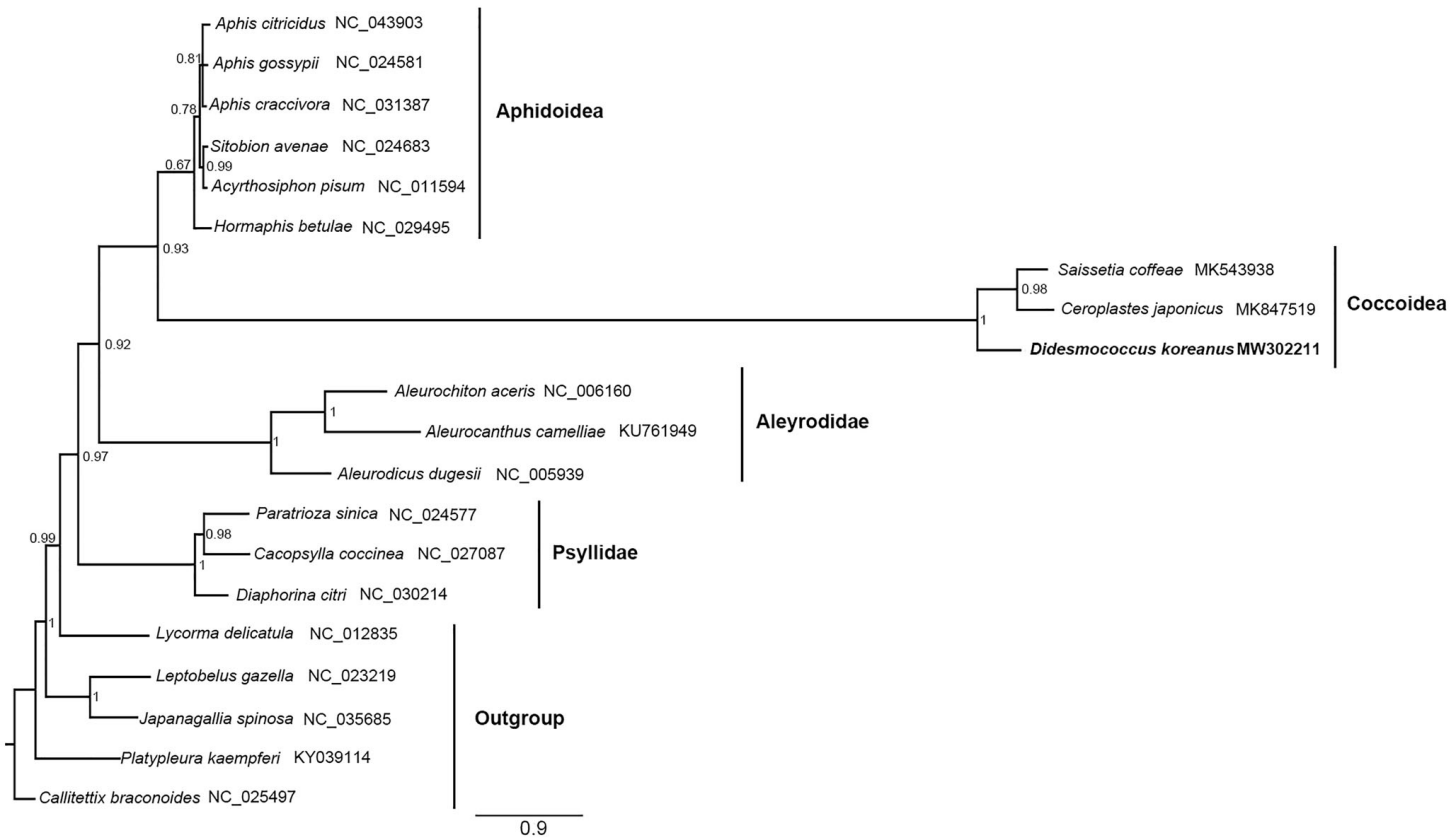
Phylogenetic tree inferred from Bayesian analysis of the nucleotide of the 13 PCGs and 2 rRNA genes. The nodal numbers indicate the posterior possibility. Genbank accession numbers for the sequences are indicated next to the species names. The newly sequenced species are indicated in bold.

## Data Availability

The data that support the findings of this study are openly available in GenBank at nucleotide database, https://www.ncbi.nlm.nih.gov/nuccore/MW302211, Associated BioProject, https://www.ncbi.nlm.nih.gov/bioproject/PRJNA698787, BioSample accession number at https://www.ncbi.nlm.nih.gov/biosample/SAMN17517890 and Sequence Read Archive at https://www.ncbi.nlm.nih.gov/sra/SRR13609017.
